# Detection of Helicobacter pylori by invasive tests in adult dyspeptic patients and antibacterial resistance to six antibiotics, including rifampicin in Turkey. Is clarithromycin resistance rate decreasing?

**DOI:** 10.3906/sag-2101-69

**Published:** 2021-06-28

**Authors:** Mustafa AKAR, Fuat AYDIN, Tuba KAYMAN, Seçil ABAY, Emre KARAKAYA

**Affiliations:** 1 Department of Gastroenterology, Bursa Yüksek İhtisas Trainig and Research Hospital, University of Health Sciences, Bursa Turkey; 2 Department of Microbiology, Faculty of Veterinary Medicine, Erciyes University, Kayseri Turkey; 3 Department ofMedical Microbiology, Şişli Hamidiye Etfal Training and Research Hospital University of Health Sciences, İstanbul Turkey

**Keywords:** Antibacterial resistance, clarithromycin, *Helicobacter pylori*, invasive test, rifampicin, Turkey

## Abstract

**Background/aim:**

The prevalence of *Helicobacter pylori* is reported to be roughly 80% in Turkey, and only very few culture-based studies are available on antibacterial resistance in adult dyspeptic patients. This study was carried out in adult dyspeptic patients with an aim to: (i) detect *H. pylori* by invasive tests (culture, polymerase chain reaction, and histopathology) and (ii) determine the current resistance rates of *H. pylori* isolates to six antibiotics, including rifampicin.

**Materials and methods:**

This study was conducted in 422 adult dyspeptic patients. The presence of *H. pylori* was demonstrated by culture, polymerase chain reaction, and the histopathology of gastric biopsy material. Antibacterial susceptibility was determined with the E-test.

**Results:**

The mean age of the patients was 50 ± 15 (range 18–90), and 265 (63%) of them were female. By culture, polymerase chain reaction, and histopathology, the presence of *H. pylori* was detected at rates of 35% (148/422), 67% (281/422), and 53% (224/422), respectively. The prevalence of *H. pylori* was determined as 75.6% (319/422). Metronidazole, levofloxacin, clarithromycin, and rifampicin resistance rates were 62%, 36%, 19%, and 12%, respectively. Monodrug, dual-drug, and multidrug resistance rates were ascertained as 36.9%, 29.4%, and 10.5%, respectively. All of the isolates were susceptible to amoxicillin and tetracycline.

**Conclusion:**

This study revealed the current prevalence of *H. pylori* in adult dyspeptic patients as 75.6%, and thereby, showed that infection with this pathogen remains highly prevalent. Although resistance to metronidazole and levofloxacin has increased over time, clarithromycin resistance rate has decreased. The high levels of resistance to metronidazole and levofloxacin limit the empirical use of these antibiotics in the eradication protocol. Owing to the low level of resistance determined for rifampicin, this antibiotic could be included in the eradication protocol, in the event of the need for rescue therapy in Turkey.

## 1. Introduction

*Helicobacter pylori* is a gram-negative microaerophilic bacterium acquired orally during childhood, and is known to colonize the gastric mucosa [1,2]. Being estimated to have colonized almost half of the world population, this pathogen may cause gastritis, peptic ulcer, gastric malignancies, immune thrombocytopenic purpura, iron deficiency anaemia, and vitamin B12 deficiency. Thus, *H.pylori* is considered a major public health concern [3–6]. The Maastricht IV and V/Florence Consensus Reports suggest that, to succeed in managing the diseases mentioned above, the presence of *H. pylori* s hould be investigated and eradication treatment should be instituted [4,6]. Furthermore, the Kyoto Global Consensus Report highlights that *H. pylori* eradication could prevent the aforementioned diseases, and also describes gastric colonisation with this bacterium as an infectious disease, irrespective of the clinical signs and symptoms observed [7].

*H. pylori* can be detected by several invasive methods, including culture, polymerase chain reaction (PCR), the rapid urease test, and histopathological examination, or by noninvasive methods such as the *H. pylori* s tool antigen test, urea breath test (UBT), and serological tests [8–11].

To date, a global standard treatment protocol has not been established for *H. pylori* . Therefore, treatment is based on the combined administration of empirically selected two or three antibiotics and the use of high doses of proton-pump inhibitors (PPIs). However, the success rate of this empirical treatment remains unsatisfactory due to increased levels of multidrug resistance [12].

As resistance to the antibiotics used for the eradication of *H. pylori* is increasing at a global level and eventually hinders treatment, it is suggested that the up-to-date susceptibility status of these antibiotics be determined by culture and an eradication programme be developed accordingly in each country [13].

In Bursa province, where the present study was conducted, the preceding investigation of the antibiotic resistance of *H. pylori* isolates was performed 12 years ago [14]. To the authors’ knowledge, there is no investigation on the antibiotic resistance status of *H. pylori* in Bursa, other than the mentioned study [14]. In addition, there is no study on rifampicin (RD) resistance in *H. pylori* isolates from Turkey.

This study was carried out in adult dyspeptic patients with an aim to: (i) detect *H. pylori* by invasive tests (culture, polymerase chain reaction, and histopathology), and (ii) determine the current resistance rates of *H. pylori* isolates to six antibiotics, including rifampicin.

## 2. Material and methods

### 2.1. Patients

In total 422 adult dyspeptic patients were enrolled in this cross-sectional study. These patients underwent upper gastrointestinal (GI) endoscopy under sedation at the Department of Gastroenterology, Bursa Yüksek İhtisas Training and Research Hospital, University of Health Sciences, Turkey, from March 2018 to September 2019. The adult patients included in this study were aged 18 and over, presented with dyspeptic symptoms (postprandial fullness, early satiation, epigastric pain, and epigastric burning) and were referred for upper GI endoscopy. Patients, known to be pregnant or diagnosed with coagulation disorders and to have received prior eradication treatment for *H. pylori* or undergone gastric surgery, were excluded from the study. Patients with a medical history of antibiotic use in the last 6 weeks were also excluded. Demographic and endoscopic data were recorded for all patients. All participants provided written informed consent before participating in the study. This study was approved by the Local Research Ethics Committee of Erciyes University Medical Faculty and designed in accordance with the 2013 Brazil version of the Helsinki Declaration. The Ethics Committee reference number is 2018/135.

### 2.2. Gastric biopsy specimens

The upper GI endoscopy procedure involved the collection of four gastric biopsy specimens from each patient, including two from the antrum and two from the corpus. Two of the specimens (1 antrum, 1 corpus) were transferred into 10% formalin solution and used for histopathological examination. The other two specimens were transferred into sterile Eppendorf tubes, containing 500 µL of brain heart infusion (BHI) broth (CM1135B, Thermo Fisher Scientific, Waltham, MA, USA), for bacteriological and molecular analyses. Histopathological examination was performed at the Department of Pathology, Bursa Yüksek İhtisas Training and Research Hospital, University of Health Sciences. Bacteriological and molecular analyses were conducted at the Department of Microbiology, Faculty of Veterinary Medicine, Erciyes University.

### 2.3. Histopathological examination

The biopsyspecimens were fixed in 10% formalin solution and embedded in paraffin. The sections cut from the paraffin blocks were stained with haematoxylin and eosin (H&E) and examined by an experienced pathologist [15].

### 2.4. Culture

The biopsy specimens maintained in BHI broth (CM1135B, Thermo Fisher Scientific) were ground using a sterile glass rod and homogenized. Twenty microliters of this material was inoculated onto Columbia blood agar base (CM0331B, Thermo Fisher Scientific) enriched with 10% defibrinated horse blood and Dent supplement (SR0147E, Thermo Fisher Scientific). The inoculated plates were incubated at 37 °C in a microaerobic atmosphere (Anaerocult C, Merck Millipore, Darmstadt, Germany) for 7–10 days. At the end of the incubation period, the *H. pylori* -suspect colonies (S type, gram-negative, and oxidase-, catalase-, and urease-positive) were assessed and their pure cultures were prepared [16,17]. The pure cultures of the isolates were stored in BHI broth supplemented with 15% glycerol at –84 °C for further use in molecular analyses and antibacterial susceptibility tests.

### 2.5. Molecular analysis

a) **DNA extraction**

Commercial microbial DNA isolation kits were used to extract DNA from both the biopsy samples (Roche high pure PCR Template Preparation Kit, Mannheim, Germany) and the *H. pylori* isolates obtained from culture (DNeasy UltraClean Microbial Kit, Qiagen, Hilden, Germany). The extraction procedures were performed in accordance with the manufacturer’s instructions.

b) **Polymerase chain reaction (PCR)**

The PCR method was used to demonstrate the presence of *H. pylori* in the gastric biopsy specimens and to confirm the identification of the isolates recovered from the selective agar as *H. pylori* by phenotypic tests. For this purpose, primers (glmM-F 5’-AAGCTTTTAGGGGTGTTAGGGGTTT-3’ and glmM-R 5’-AAGCTTACTTTCTAACACTAAC GC-3’) specific to the phosphoglucosamine mutase (glmM) gene of *H. pylori* were used, and the test was performed by a method of Lu et al. [18]. The amplification cycles consisted of denaturation at 93 °C for 1 min, primer annealing at 58 °C for 1 min, and extension at 72 °C for 1 min. Samples were amplified through 35 cycles. Amplified products were resolved by gel electrophoresis on 1.5% agarose (Biomax, Agarose, Lot no. 124543PR, from Prona, European Economic Community) and visualized under a UV transilluminator (G:BOX Chemi XRQ; Syngene, Cambridge, UK). Bands, which were 294-bp in size, were considered as a positive result.

### 2.6. Antibacterial susceptibility testing

The minimal inhibitory concentration (MIC) values of amoxicillin, clarithromycin, levofloxacin, metronidazole, rifampicin, and tetracycline against the *H. pylori* isolates were determined with the E-test. Mueller–Hinton agar supplemented with 7% defibrinated horse blood (R54092, Thermo Fisher Scientific) was used for this purpose. Accordingly, a suspension prepared from 3-day-old *H. pylori* cultures (Mac Farland No:3) was spread onto Mueller–Hinton agar (CM0337B, Thermo Fisher Scientific) supplemented with 7% defibrinated horse blood, and after the agar surface dried, the E-test (The Liofilchem MIC Test Strips, Italy) strips of the selected antibiotics were placed onto the agar. The plates were incubated at 37 °C in a microaerobic environment for 48–72 h [16]. The results were evaluated according to the criteria of the European Committee on Antimicrobial Susceptibility Testing (EUCAST) [19]. The MIC values (mg/L) of amoxicillin, clarithromycin, tetracycline, metronidazole, levofloxacin, and rifampicin were≤0.125, ≤0.5, ≤1, ≤8, ≤1, and ≤1, respectively. Isolates, which were resistant to three or more antibiotics, were considered to be multidrug resistant.

### 2.7. Standard strain

*Helicobacter pylori* ATCC 700824 was used as a positive control in culture, molecular analysis, and antibiotic susceptibility tests.

### 2.8. Criterion used to detect the presence of H. pylori

*H. pylori* was considered to be present in the samples, when the result of any of the invasive tests (culture, PCR, and histopathology) was positive.

### 2.9. Statistical analysis

Data were analysed using the SPSSversion 20.0 (IBM Corporation, Armonk, NY, USA). Values were expressed as mean ± standard deviation for normally distributed variables, and as count and percent for categorical variables.

## 3. Results

### 3.1. Clinical data

In total, 422 adult dyspeptic patients were included in this study. The mean age of the patients was 50 ± 15 years (range: 18–90) and 265 (63%) of them were female. The most common endoscopic finding was gastritis, accounting for 62%, followed by reflux esophagitis (16%), duodenal ulcer/duodenitis (15%), benign gastric ulcer (4.5%), normal gastric mucosa (2%), and gastric malignancy (0.5%) (Table 1). Gastritis, benign gastric ulcer, normal gastric mucosa, and gastric malignancy were also demonstrated histopathologically.

**Table 1:** Demographic and clinical data of the patients (n: 422).

Characteristics		n (%)
Age (mean ± SD)		50 ± 15
Sex		
	Male	157 (37)
	Female	265 (63)
Endoscopic findings		
	Gastritis	261 (62)
	Reflux esophagitis	68 (16)
	Duodenal ulcer/duodenitis	62 (15)
	Gastriculcer	19 (4.5)
	Normal	10 (2)
	Gastric malignancy	2 (0.5)

SD: Standard deviation.

### 3.2. Prevalence of H. pylori and results of the invasive tests

**Figure 1:**
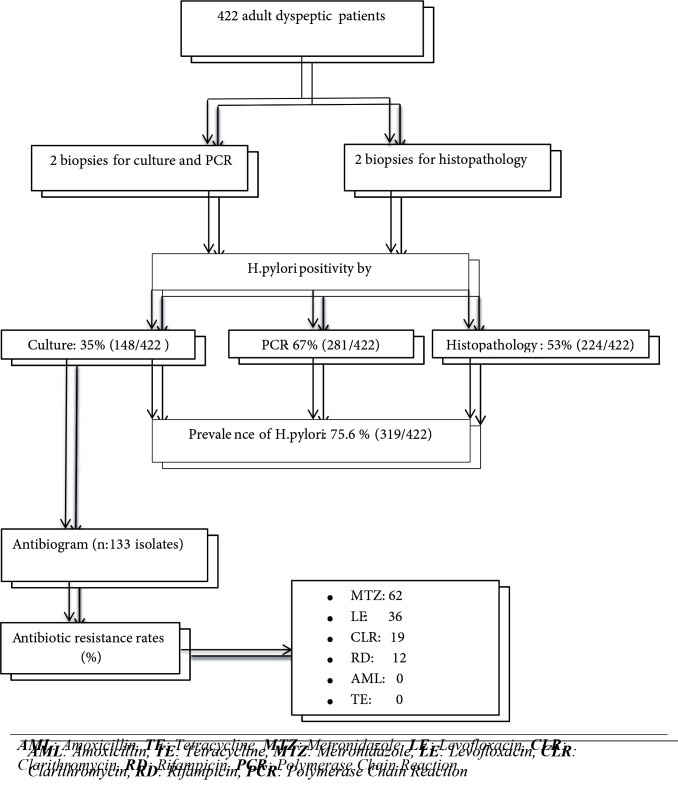
Flow diagram of the current study.

**Figure 2:**
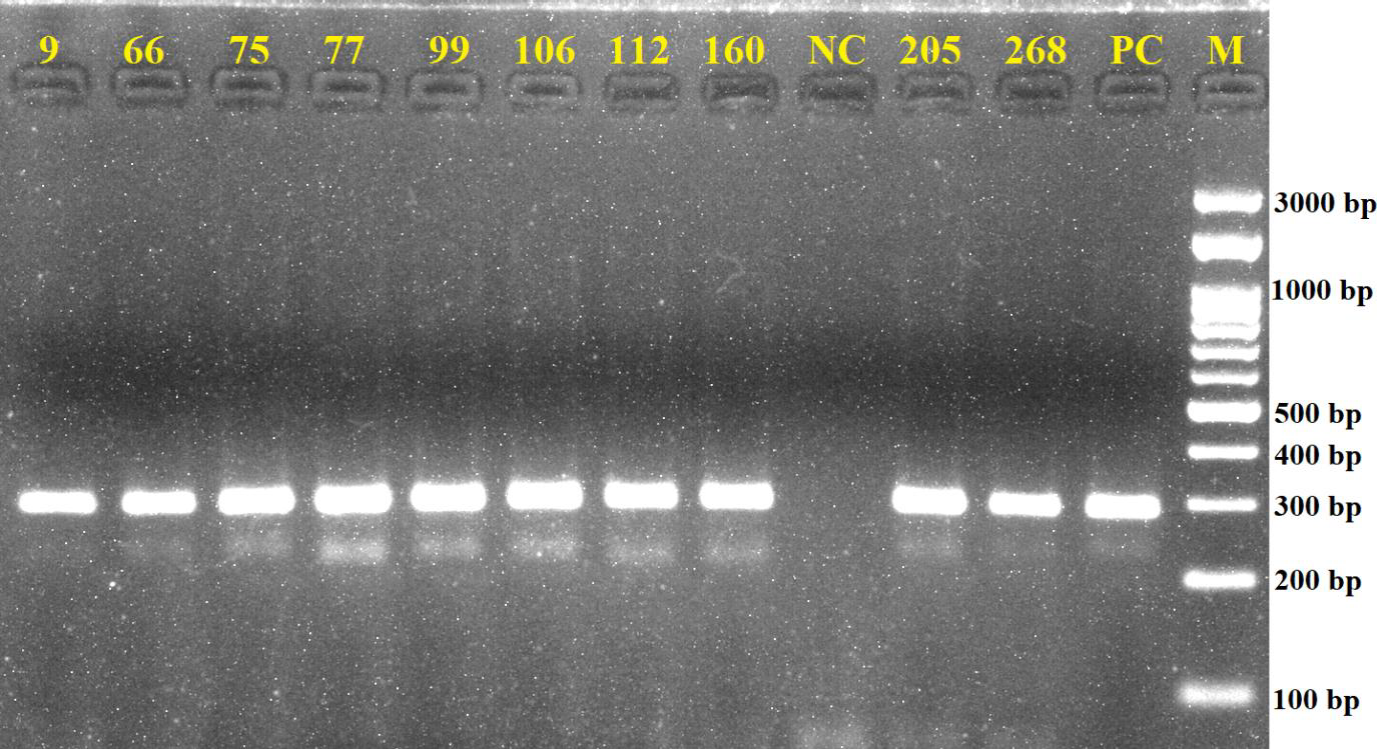
Agarose gel electrophoresis of PCR products obtained by using primer pairs glmM-F and glmM-R (297bp). NC: Negative control (distilled water), PC: Positive control (H. pylori, ATCC 700824), Representative positive samples (9, 66, 75, 77, 99, 106, 112, 160, 205, 268): H. pylori isolates recovered from gastric biopsies in the current study M: Marker, 100bp DNA Ladder H3 RTU, GeneDireX.

The prevalence of *H. pylori* was determined as 75.6% (319/422). The presence of *H. pylori* was detected at rates of 35% (148/422), 67% (281/422), and 53% (224/422) by culture, PCR, and histopathological examination, respectively (Figure 1). A representative image of the agarose gel electrophoresis of the PCR products is shown in Figure 2.

### 3.3. Antibacterial susceptibility testing

**Figure 3:**
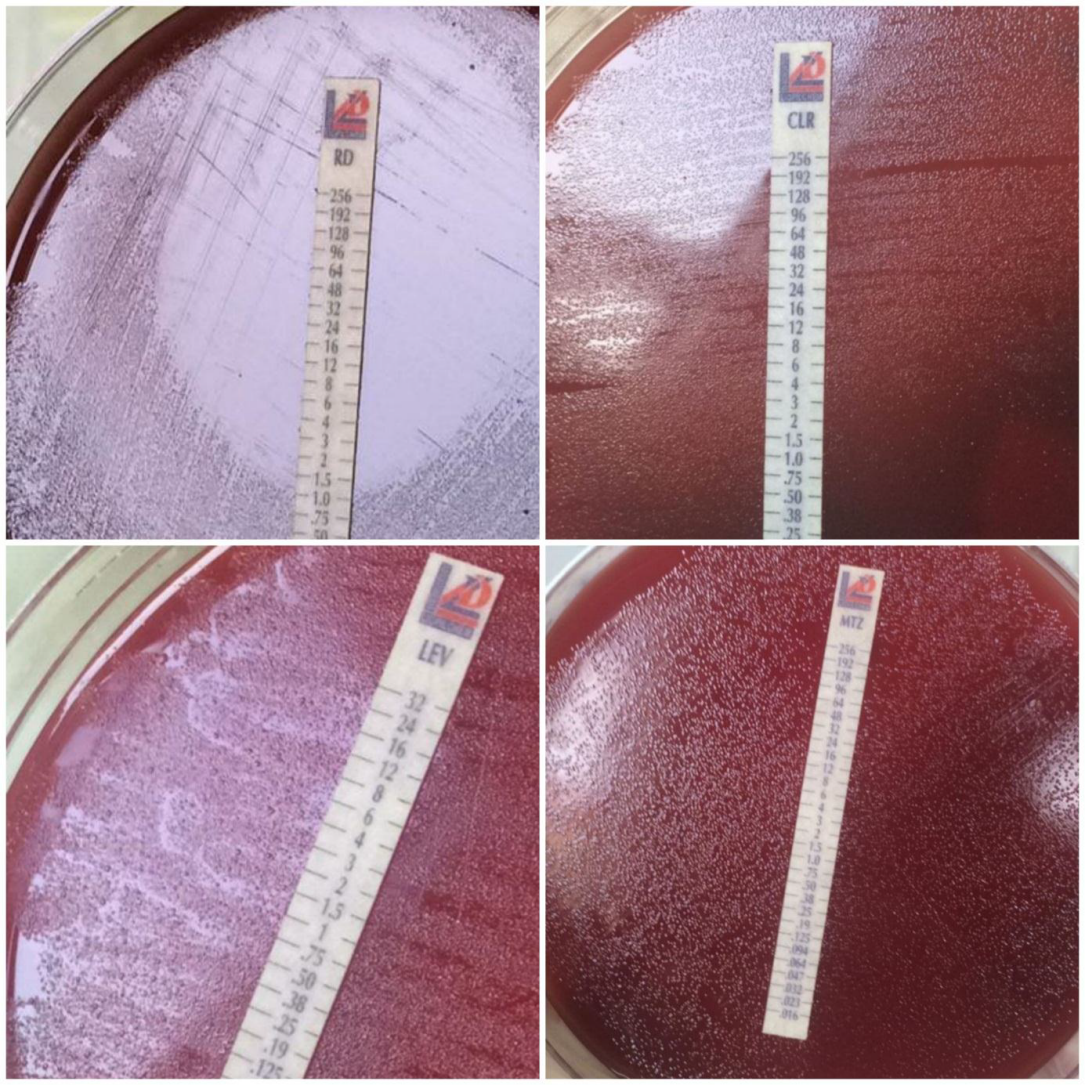
Representative images regarding E-test rifampicin, clarithromycin, levofloxacin, and metronidazole.

Out of the 148 *H. pylori* isolates recovered from culture, 15 could not be subcultured with passages. Thus, 133 isolates were tested for antibacterial susceptibility. Based on the test results, all 133 (100%) isolates were susceptible to amoxicillin and tetracycline. Metronidazole, levofloxacin, clarithromycin, and rifampicin resistance were determined at levels of 62%, 36%, 19%, and 12%, respectively (Figure 1). A representative image of the E-test is shown in Figure 3. A detailed assessment of the test results demonstrated that, out of the 133 isolates, 31 (23.3%) were susceptible to all of the antibiotics tested, 49 (36.9%) were resistant to a single antibiotic, 39 (29.4%) were resistant to two antibiotics, and 14 (10.5%) were multidrug resistant (Table 2).

**Table 2:** Antibiotic susceptibility profiles of the H. pylori isolates (n: 133).

Profiles		n (%)
No resistance to all drugs		31 (23.3)
Monodrug resistance		49 (36.9)
MTZ LE CLR RD		37 (27.8)9 (6.8)1 (0.8)2 (1.5)
Dual-drugs resistance		39 (29.4)
	MTZ+LEMTZ+CLRMTZ+RDLE+CLRLE+RD	21 (15.8)6 (4.5)6 (4.5)5 (3.8)1 (0.8)
Multidrugs resistance		14 (10.5)
	Triple resistance	12 (9.1)
	MTZ+LE+CLRMTZ+LE+RDMTZ+CLR+RDLE+CLR+RD	7 (5.3)1 (0.8)2 (1.5)2 (1.5)
	Quadruple resistance	2 (1.5)
MTZ+LE+CLR+RD		2 (1.5)

MTZ: Metronidazole, LE: Levofloxacin, CLR: Clarithromycin, RD: Rifampicin.

## 4. Discussion

To the authors’ knowledge, the present study is the most comprehensive molecular and culture-based research conducted to date on *H.pylori* in Turkey. Furthermore, this study is the first to report rifampicin resistance in *H.pylori* isolates from Turkey. 

The prevalence of *H. pylori* varies greatly in developed and developing countries (18.9–87.7%) [20]. In a community-based survey conducted in 4663 adults in Turkey, the prevalence of *H. pylori* was determined as 82.5% with the UBT [21]. The prevalence of *H. pylori* in dyspeptic patients is reported to range between 17% and 34% in developed countries [22,23], 75.6% and 86% in developing countries [24,25], and 59.6% and 71.3% in Turkey [26,27]. In the present study, the prevalence of *H. pylori* (75.6%) was higher than the prevalence reported for developed countries and similar to that reported for developing countries. 

As is the case throughout the world, in Turkey, the antibiotics most commonly used for the eradication of *H. pylori* are clarithromycin, metronidazole, levofloxacin, tetracycline, and amoxicillin [28–31]. Rifampicin is generally used for the treatment of tuberculosis, therefore, in order to prevent the development of resistance, this antibiotic is purposefully not used for the empirical treatment of *H. pylori* in Turkey.

Reports have shown that the resistance of *H. pylori* to these six antibiotics varies among both countries and regions [12,13,32–35]. In most of the WHO regions, clarithromycin, metronidazole, and levofloxacin resistance rates of >15% have been reported [13,36]. Furthermore, tetracycline and amoxicillin resistance rates have been reported to be <10% across the globe [36,37]. It has also been reported that, rifampicin resistance ranges between 0% and 33% [16,36,38–40]. In addition, clarithromycin, metronidazole, levofloxacin, tetracycline, and amoxicillin resistance rates range between 18% and 34%, 28.7% and 56%, 0% and 22%, 0% and 10%, and 0% and 14%, respectively, in different regions of the world [13,16,39–41], and are 3.6% and 28.1%, 33.8% and 64.9%, 8% and 21.9%, 16.1%, and 20.7%, respectively, in the neighbouring countries of Turkey [38,40,42,43].

According to research conducted in different regions of Turkey in the past decade, clarithromycin, metronidazole, levofloxacin, tetracycline, and amoxicillin resistance rates range between 18.1% and 38.1%, 31.1% and 45.5%, 18.2% and 34%, 0% and 9.1%, and 0% and 2.9%, respectively [27,44–46]. In a previous study carried out in Bursa province, which is also the location of the present study, the rates of clarithromycin, metronidazole, tetracycline, and amoxicillin resistance were found to be 41.9%, 41.9%, 3.2%, and 3.2%, respectively [14] (Table 3).

**Table 3:** H. pylori antibiotic resistance rates among adults in recent years in different regions of Turkey.

Study year	Method	Number of patients	Number of isolates	Region(city)	Resistance rate (%)							Ref.
					AML	TE	MTZ		CLR	LE	RD	
2008	E-test	31	31	West (Bursa)	3.2	3.2		41.9	41.9	-	-	14
2010	E-test	149	102	South (Mersin)	0	9.1		45.5	18.1	18.2	-	46
2011	E-test	344	104	North (Trabzon)	2.9	1		31.1	28.2	34	-	27
2013	E-test	98	98	West (İstanbul)	0	0		35.5	36.7	29.5	-	44
2017	E-test	63	63	West (İstanbul)	-	-		-	38.1	-	-	45
This study	E-test	422	133	West (Bursa)	0	0		62	19	36	12	-

AML: Amoxicillin, TE: Tetracycline, MTZ: Metronidazole, LE: Levofloxacin, CLR: Clarithromycin, RD: Rifampicin, Ref.: Reference.

The present study demonstrated that clarithromycin resistance (19%) had decreased over time in Turkey, and this was attributed to the clinical use of clarithromycin having decreased. On the other hand, metronidazole (62%) and levofloxacin (36%) resistance were ascertained to have increased in Turkey. This could be attributed to the increased clinical use of this antibiotic as well as to patients not adhering to the prescribed administration dose and period. In view of the high levels of clarithromycin, metronidazole, and levofloxacin resistance detected in Turkey, it is suggested that the empirical use of these antibiotics for the eradication of *H. pylori* should be avoided, and their clinical use should be based on antibacterial susceptibility test results. In addition, we determined that all of the tested *H. pylori* isolates were susceptible to tetracycline and amoxicillin. Therefore, it is considered that both tetracycline and amoxicillin can be safely used for the empirical treatment of *H. pylori* in Turkey. To the authors’ knowledge, rifampicin resistance has not been investigated before in *H. pylori* isolates from Turkey. The present study reports, for the first time, the resistance level of *H. pylori* to rifampicin as 12%. The level of resistance to rifampicin is lower than the resistance levels to the antibiotics used for the eradication of *H. pylori* in Turkey, including clarithromycin, metronidazole, and levofloxacin. This result suggests that, rifampicin could be included in the eradication protocol of *H. pylori* , when salvage therapy is needed in Turkey.

Worldwide, the multidrug resistance rate of *H. pylori* is reported as 9.6% [37]. In the present study, the rate of resistance to at least one antibiotic was ascertained as 76.6% and multidrug resistance was determined at a rate of 10.5% (Table 2). Therefore, to achieve success in the eradication of *H. pylori* , it is required to determine the current antibiotic resistance status, and to establish appropriate eradication protocols in view of this status.

### 4.1. Limitations of the study

i-) The current study is monocentric, so the results obtained may not accurately represent the entire territory of Turkey.

ii-) This study does not include a sufficient number of patients to determine the exact prevalence of *H. pylori* in adult dyspeptic patients.

## 4. Conclusion

The prevalence of *H. pylori* in adult dyspeptic patients was found to be higher than that in developed countries and similar to that in developing countries. Resistance to metronidazole and levofloxacin was determined to have increased over time. Although resistance to clarithromycin had decreased over time, it was observed to remain above 15%. It is considered that the high levels of resistance detected in *H. pylori* isolates to metronidazole and levofloxacin would constitute a major problem in empirical treatment. On the other hand, as no resistance was detected to amoxicillin and tetracycline, these antibiotics were considered to be safe for use in the eradication of *H. pylori.* Furthermore, in view of the low level of resistance to rifampicin, this antibiotic could be included in the eradication protocol of *H. pylori* in Turkey as an option for rescue therapy.

## Disclaimers/conflict of interest

The manuscript has not been published previously elsewhere. There is no specific funding has been received to report this submission. All of the authors declare that they have all participated in the design, execution, and analysis of the paper, and that they have approved the final version. All authors are in agreement with the content of the manuscript. The authors have no conflict of interest to disclose.
